# Visualizing domain wall and reverse domain superconductivity

**DOI:** 10.1038/ncomms5766

**Published:** 2014-08-28

**Authors:** M. Iavarone, S. A. Moore, J. Fedor, S. T. Ciocys, G. Karapetrov, J. Pearson, V. Novosad, S. D. Bader

**Affiliations:** 1 Department of Physics, Temple University, Philadelphia, Pennsylvania 19122, USA; 2 Department of Physics, Drexel University, Philadelphia, Pennsylvania 19104, USA; 3 Materials Science Division, Argonne National Laboratory, Argonne, Illinois 60439, USA; 4 Present address: Institute of Electrical Engineering, Slovak Academy of Sciences, Dúbravská cesta 9, 84104 Bratislava, Slovakia

## Abstract

In magnetically coupled, planar ferromagnet-superconductor (F/S) hybrid structures, magnetic domain walls can be used to spatially confine the superconductivity. In contrast to a superconductor in a uniform applied magnetic field, the nucleation of the superconducting order parameter in F/S structures is governed by the inhomogeneous magnetic field distribution. The interplay between the superconductivity localized at the domain walls and far from the walls leads to effects such as re-entrant superconductivity and reverse domain superconductivity with the critical temperature depending upon the location. Here we use scanning tunnelling spectroscopy to directly image the nucleation of superconductivity at the domain wall in F/S structures realized with Co-Pd multilayers and Pb thin films. Our results demonstrate that such F/S structures are attractive model systems that offer the possibility to control the strength and the location of the superconducting nucleus by applying an external magnetic field, potentially useful to guide vortices for computing application.

## Abstract (web summary)

When superconductivity emerges in a thin superconductor grown on a ferromagnet, it does so in an array of interacting superconducting and normally conducting channels. Maria Iavarone and colleagues use scanning tunnelling microscopy to image how these channels form and interact.

## Graphical abstract

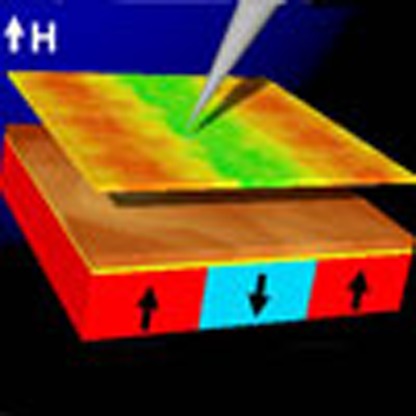

Recent experimental advances in fabrication and in the measurement of mesoscopic superconductivity have renewed theoretical interest in ferromagnet-superconductor (F/S) hybrid structures. The interaction between superconductivity and magnetism, with Cooper pairs transferred from the superconductor into the ferromagnet, leads to new physical phenomena due to the proximity effect, with the mutual influence of extended electron states shared by fundamentally different materials^1^. However, if a thin insulating layer separates the superconducting and the ferromagnetic layers, the proximity effect will be suppressed and the electromagnetic interaction will dominate. In this case, the inhomogeneous distribution of the magnetic field produced by the ferromagnet substantially modifies the nucleation of the superconducting order parameter leading to a significant change in the superconducting properties^2^.

Previous investigations of planar F/S hybrid structures focused on transport measurements^3^,^4^,^5^,^6^,^7^, vortex imaging^8^,^9^, F/S junctions^10^ or the visualization of reverse domain wall superconductivity by scanning laser microscopy with a spatial resolution of micrometres^11^,^12^. Here, we present low temperature scanning tunnelling microscopy (STM) and spectroscopy measurements focused on the direct visualization of the nucleation of superconductivity on the length scale of the superconducting coherence length. Moreover, we experimentally demonstrate that F/S structures are efficient systems to spatially manipulate mesoscopic superconductivity and guide vortices by using an external magnetic field. Prior experimental work in the field of manipulation of superconductivity showed that the polarization of a ferroelectric, much like a ferromagnet domain polarization, could be used to control the modulation of the superconducting condensate at the nanoscale^13^. On the other hand, manipulation of a single vortex has been achieved by using a magnetic force microscopy tip, with results giving an insight into pinning strength and pinning mechanism in high-temperature superconducting materials^14^.

We fabricated F/S heterostructures, composed of a superconducting layer placed on the top of a ferromagnetic layer, separated by a thin insulator (I) layer, with Co/Pd multilayer as the magnetic material and Pb thin film as the superconductor. These magnetic multilayers exhibit relatively large stray fields (compared with the critical field of the superconductor used in this study) and have striped magnetic domain patterns with the easy axis of magnetization perpendicular to the film plane^15^. The regions of the superconducting film above the domain walls experience a lower stray field, and therefore, the superconductivity is expected to nucleate at these regions when decreasing the temperature below the superconducting critical temperature *T*_c_, giving rise to an inhomogeneous superconducting state. We use STM and spectroscopy to study two F/S heterostructures (samples A and B), with ferromagnetic films that have different magnetic domain sizes.

Here we demonstrate that upon decreasing the temperature below *T*_c_, superconductivity emerges above the magnetic domain walls and is confined to length scales on the order of 100 nm in samples where the domain width of the ferromagnet is large enough compared with the characteristic nucleation length (that is, coherence length of the superconductor). The domain wall superconductivity leads to nanoscale confinement of the Cooper pairs by the inhomogeneous magnetic field. The emergence of superconductivity and the evolution of the inhomogeneous superconducting state with temperature strongly depend on the size of the magnetic domains in the ferromagnet. Moreover, upon decreasing the temperature below *T*_c_, in the presence of an applied magnetic field perpendicular to the sample surface, we observe the nucleation of superconductivity in regions above magnetic domains that are aligned antiparallel to the applied field. Reversal of the polarity of the applied field leads to a shift of the superconducting regions to oppositely polarized magnetic domains, hence the appearance of reverse domain superconductivity. Furthermore, imaging of the local density of states deep in the superconducting state reveals the presence of spontaneous vortices induced by the stray field of the ferromagnet.

## Results

### Inhomogeneous superconducting state in F/S hybrids

**Figure 1:**
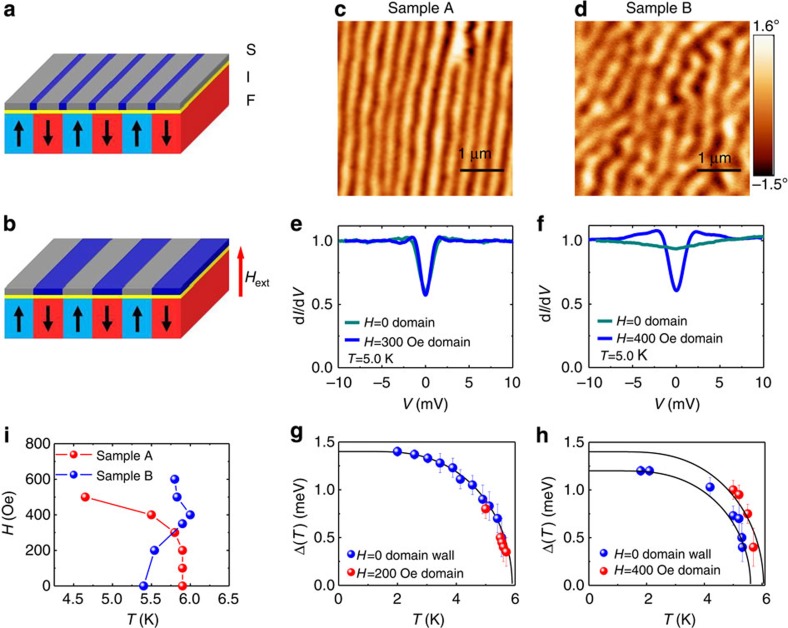
Phase diagram of (Co-Pd)/Pb systems from tunnelling spectroscopy. (**a**,**b**) Schematics of a hybrid system, consisting of a ferromagnet (F), insulator (I) and a superconducting film (S), close to the superconducting critical temperature. (**a**) In zero applied magnetic field, the superconductivity nucleates in the regions above the domain walls (shown in blue). (**b**) In the presence of an applied magnetic field that compensates one type of domain, the superconductivity nucleates above the antiparallel domains (blue regions). (**c**,**d**) Magnetic force microscopy phase images (scan area of 4 × 4 μm^2^, scale bar, 1 μm) at room temperature of two ferromagnetic samples used in this experiment. MFM measurements have been performed after deposition of the Al_2_O_3_ layer. (**c**) Sample A (200 bilayers of Co (2 nm)/Pd (2 nm)) showing a stripe domain pattern with average domain size *w*≈200 nm. (**d**) Sample B (50 bilayers of Co (2 nm)/Pd (2 nm)) showing a stripe domain pattern with average domain size *w*≈300 nm. (**e**,**f**) STM conductance spectra acquired on (**e**) (Co-Pd)/Pb sample A and (**f**) (Co-Pd)/Pb sample B, at a position corresponding to the middle of a magnetic domain. The two spectra were acquired in the absence and in the presence of an external field *H* at *T*=5.0 K. The tunnelling spectra have been normalized to the conductance value at *V*=−10 mV. (**g**,**h**) Temperature dependence of the gap values obtained from the conductance spectra acquired on the (**g**) (Co-Pd)/Pb sample A and (**h**) (Co-Pd)/Pb sample B. The blue dots represent the temperature dependent gap values at the location above a magnetic domain wall in the absence of an external field. The red dots represent the temperature dependent gap values at a location corresponding to the middle of the magnetic domain in the presence of a compensating applied magnetic field. The gap values have been derived by the Bardeen-Cooper-Schrieffer (BCS) DOS fitting. The error bars represent the standard deviation obtained from the fit. The experimental points are compared with the BCS gap equation Δ(*T*) (solid lines). (**i**) Phase diagram of the highest local *T*_c_ versus *H* derived from local tunnelling spectroscopy measurements for both samples.

We fabricated two F/S heterostructures (samples A and B) with different magnetic domain sizes (schematics are shown in Fig. 1a,b). Sample A consists of 200 bilayers of Co (2 nm)/Pd (2 nm) as the ferromagnet and a 30-nm film of Pb as the superconductor. A 10-nm Al_2_O_3_ film was used as an insulator between the superconductor and the ferromagnet to suppress the proximity effect. The ferromagnet, in this sample, has an average domain size of *w*≈200 nm as seen in the magnetic force microscopy (MFM) image shown in Fig. 1c. On the other hand, sample B consists of a ferromagnetic film with 50 bilayers of Co (2 nm)/Pd (2 nm) resulting in an average domain size *w*≈300 nm as shown in Fig. 1d, and a 30-nm film of Pb as superconductor. It is important to note that the magnetic domains in both samples are independent of the applied magnetic field in the range of fields considered in this paper (as shown in Supplementary Fig. 1). Therefore, we can conclude that no domain wall motion is taking place during STM measurements in applied magnetic field for the range of fields explored.

The Pb films were grown on the Al_2_O_3_-coated ferromagnet in an ultrahigh vacuum (UHV) system at base pressure 10^−11^ Torr in a dedicated chamber linked to the cryogenic STM, thus avoiding any exposure of the Pb film to air. The choice of superconducting material was dictated by the value of the upper critical field of lead (*H*_c2_(*T*=1.5 K)≈1,200 Oe) that is relatively low compared with the stray fields of the Co/Pd multilayers, thus providing a wide region of the *H-T* phase diagram where superconductivity can be spatially modulated by the stray fields of the underlying magnetic pattern. A reference Pb film was deposited on Al_2_O_3_, without ferromagnet for comparison and it was characterized by STM (see Supplementary Fig. 2).

The spatial distribution of the surface magnetic field induced by the ferromagnet in the F/S hybrid system depends upon the ratio of two length scales: the thickness of the ferromagnet *D,* and the domain width *w*^16^. In the case of both sample A (*D*>*w*) and sample B (*D*≈*w*), there is a gradient of stray field above each domain with the field reaching the maximum value at the centre of the domain (Supplementary Fig. 3 and Supplementary Note 1). If the thickness of the superconductor is less than the superconducting penetration depth (in the case of bulk Pb *λ*=38 nm^17^), the magnetic field distribution will not be affected by the superconducting transition, as in our case^16^. When the temperature is decreased below *T*_c_, the superconductivity is expected to first nucleate at the location where the stray field is minimum, that is, at the domain wall, which is a realization of domain wall superconductivity^2^ (Fig. 1a). The theoretical condition for the nucleation of the superconductivity at a domain wall is that the characteristic length scale for the nucleation of superconductivity, that is, the coherence length at the superconducting critical temperature of the S/F system *ξ*(*T*_c_), should be smaller than the domain half-width to avoid overlap of the superconducting nuclei^7^. The local *T*_c_ value above the domains will be lower than the *T*_c_ above the domain wall. On the other hand, when an external magnetic field is applied perpendicular to the film, the regions with minimum magnetic field will shift to a different position due to magnetic field compensation effect (Fig. 1b). Therefore the applied magnetic field will spatially shift the superconducting nucleus to the centre of the compensated domain, as shown schematically in Fig. 1b.

Systems with purely electromagnetic interactions can be described phenomenologically using the Ginzburg–Landau and London formalisms. Aladyshkin *et al.*^18^,^19^ solved the linearized Ginzburg–Landau equations for a superconducting film and a ferromagnetic film with a periodic one-dimensional domain structure with magnetization perpendicular to the film surface. The spatial inhomogeneity of the stray field leads to different regimes of the order parameter nucleation and an unusual phase boundary *T*_c_(*H*) depending upon parameters, such as the stray field values, characteristic length scale for nucleation of the superconductivity and magnetic domain size.

Local differential tunnelling conductance data (*dI*/*dV*), obtained from the scanning tunnelling spectroscopy measurements, provide information about the local density of states (LDOS) in the material. Mapping of the LDOS is an effective method of visualizing a temperature and magnetic field evolution of the inhomogeneous superconducting state on the nanometre scale. In sample B, containing wider magnetic domains, the superconducting inhomogeneities are more strongly pronounced than in sample A (as shown in Supplementary Figs 4 and 5 and Supplementary Note 2). In sample A, close to *T*_c_ and at *H*=0 Oe, the tunnelling spectra are spatially homogeneous and do not depend on the distance from the magnetic domain wall (Supplementary Fig. 4). Moreover, spectra acquired at positions corresponding to the middle of a magnetic domain do not change in external fields up to *H*=300 Oe (Fig. 1e). On the other hand, in sample B under the same conditions we observe superconducting gap-like spectra with a pronounced dip in the LDOS at the Fermi energy *E*_F_ (zero bias conductance, ZBC) and symmetric coherence peaks at positive and negative energies from the Fermi energy. In the other locations, the spectra look similar to the ones observed in the normal state (Supplementary Fig. 5). Application of a field of *H=*+400 Oe leads to even more inhomogeneous superconducting state, with the strongest superconductivity now observed at the locations that were normal at *H*=0 Oe (Fig. 1f). These locations correspond to the centres of magnetic domains that are antiparallel to the applied field. The local stray field is reduced here as a consequence of the compensation by the applied field. Such a strong spatial variation of superconductivity in sample B is in stark contrast to the state of sample A, in which no spatial variation of the local *T*_c_ is observed up to applied fields of *H*=200 Oe (Fig. 1g). In sample B we find a strong magnetic field enhancement of the superconductivity at the centres of domains that are antiparallel to the applied field, with local *T*_c_ values that exceed any observed at *H*=0 Oe (Fig. 1h–i). In Fig. 1h we compare the superconducting gap values (obtained from the tunnelling spectra) in sample B at *H*=0 Oe in locations above the magnetic domain wall and at *H=*+400 Oe in locations above the centre of an antiparallel domain. The data show that the *T*_c_ value is location dependent. A comparison of the superconducting phase diagrams *T*_c_(*H*) for both samples is summarized in Fig. 1i. The phase diagram is obtained from the tunnelling spectra acquired as a function of applied field and temperature with each point corresponding to the highest local *T*_c_ value obtained at each field. The magnetic field enhancement of the superconductivity in sample B is shown by a pronounced re-entrant behaviour of the *H-T* phase line.

### Visualization of reverse domain superconductivity

**Figure 2:**
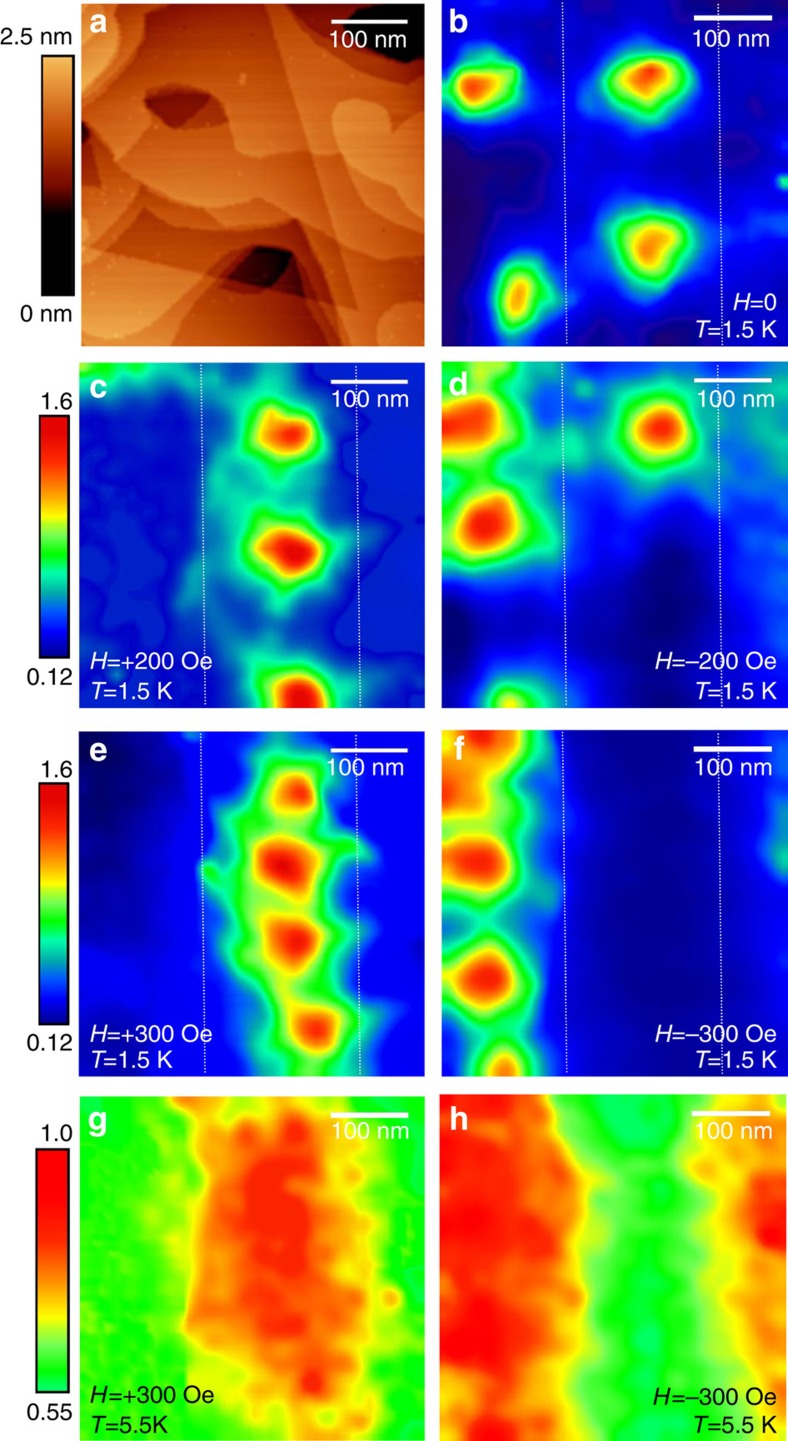
Reverse domain superconductivity in (Co-Pd)/Pb systems (sample A). (**a**) STM topography image of the Pb film in sample A. Scan area is 450 × 450 nm^2^. The tunnelling conditions are: *V*=−20 mV and *I*=100 pA. (**b**–**h**) Zero bias conductance maps acquired on the same area at different applied fields and temperatures. The white dashed lines indicate the approximate positions of the domain walls as inferred from the vortex configurations at different fields. The conductance maps are normalized to the conductance maps at *V*=−10 mV. (**b**) *H*=0 Oe, *T*=1.5 K; (**c**) *H*=+200 Oe, *T*=1.5 K; (**d**) *H*=−200 Oe, *T*=1.5 K; (**e**) *H*=+300 Oe, *T*=1.5 K; (**f**) *H*=−300 Oe, *T*=1.5 K; (**g**) *H*=+300 Oe, *T*=5.5 K; (**h**) *H*=−300 Oe, *T*=5.5 K. Scale bar, 100 nm for all images.

A map of the ZBC, that is, the LDOS at the Fermi level, reveals normal and superconducting regions with atomic resolution. Therefore, it is a direct experimental technique to visualize the emergence of domain wall superconductivity and reverse domain superconductivity with high spatial resolution. Although STM is not sensitive to the magnetic field, the presence of a superconducting film can be used as an ‘indicator’ of the underlying magnetic pattern. Local changes in tunnelling spectra performed in applied magnetic fields of opposite polarity will indeed reveal the location and polarity of the underlying magnetic domains. In Fig. 2 we present the magnetic field dependence of the ZBC maps acquired from sample A. Figure 2a is the topography image of the top Pb film, which shows atomically flat terraces. Figure 2b–h are LDOS maps at *E=E*_F_ acquired on the same area as a function of *H* and *T*. The ZBC conductance map at *T=1.5* K in zero externally applied field (Fig. 2b) reveals the presence of vortices spontaneously induced by the stray field of the ferromagnet, in agreement with theoretical predictions^20^,^21^. Vortices appear as normal regions in the conductance map, which are regions with higher ZBC. Therefore, the polarity of the vortex is indistinguishable in the STM image. By applying a magnetic field of *H=*+200 Oe and +300 Oe (Fig. 2c,e), the vortex density increases in some regions and decreases in other regions. In opposite fields of *H=−*200 Oe and −300 Oe (Fig. 2d,f), the regions where the vortex density increases and decreases are interchanged, with the vortices always following a stripe-like pattern. These images suggest that the regions with increasing vortex density in positive applied fields are above a positive magnetic domain. Instead, regions with decreasing vortex density in positive applied fields, due to compensation effects, are above a negative magnetic domain. We can also conclude that the observed vortices have opposite polarities and they are located above the corresponding domain with the same polarity^8^,^22^. The formation of vortex chains is due to confinement by the stray magnetic field from the underlying magnetic domains^9^.

Close to *T*_c_ in the presence of an applied magnetic field, superconducting regions will be located above the magnetic domains antiparallel to the external field, whereas normal regions will be located above the magnetic domains with the same polarity as the external field. In Fig. 2g,h the conductance maps at *H*=+300 Oe and *H*=−300 Oe, respectively, show the direct visualization of the reverse domain superconductivity. Here, the effect of reversing the applied field produces two complementary images with normal and superconducting regions that exchange their position upon reversal of the applied field.

### Visualization of domain wall superconductivity

**Figure 3:**
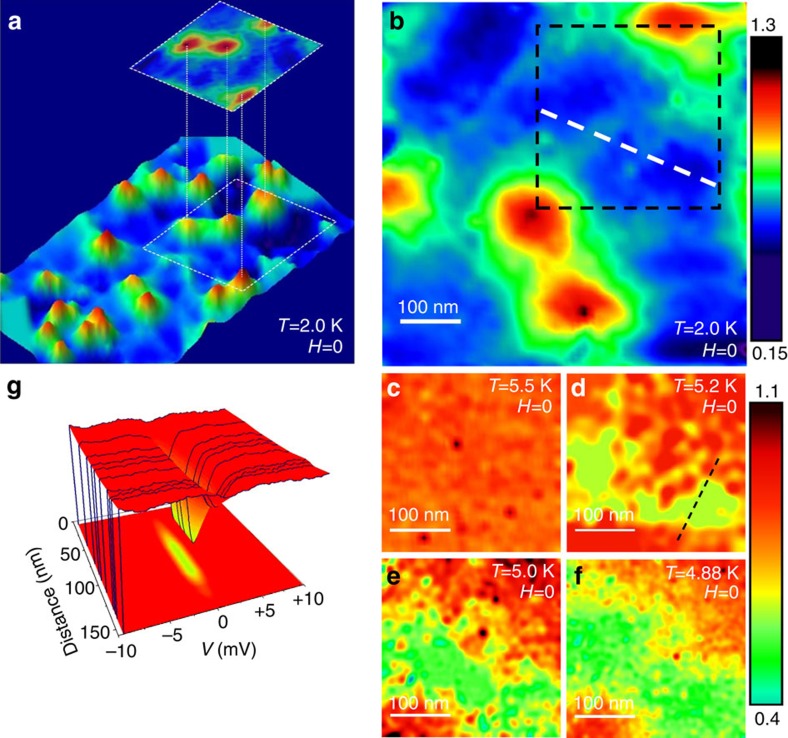
Emergence of superconductivity above a domain wall (sample B). (**a**) Zero bias conductance map acquired at 2.0 K in zero applied field for sample B. The image shows spontaneous vortices produced by the underlying magnetic pattern. Scan area is 900 × 1,625 nm^2^. The image was obtained by acquiring several images of size 600 × 600 nm^2^. The inset shows the same image reported in **b**. The image is rotated and superimposed to **a** to show the field of view of **b** on a larger map. The vertical dashed lines indicate the position of the individual vortices. The white dashed square is the scan area in **b**. (**b**) Zero bias conductance map at *T*=2.0 K and applied field *H*=0 Oe in a smaller field of view. The scanning area is 600 × 600 nm^2^, scale bar, 100 nm. The dashed square in the image shows the field of view (300 × 300 nm^2^) for the zero bias conductance images shown in **c** *T*=5.5 K, (**d**) *T*=5.2 K, (**e**) *T*=5.0 K and (**f**) *T*=4.88 K (scale bar, 100 nm for **c**–**f**). The magnetic field is *H*=0 Oe for all images. The white dashed line shows the approximate position of the domain wall. (**g**) Series of local tunnelling *dI*/*dV* spectra acquired across the dashed line in **d** at 5.2 K (tunnelling conditions *V*=−10 mV and *I*=100 pA). The conductance maps are normalized to the conductance maps at *V*=−10 mV.

In sample B the magnetic domains are wider and the magnetic pattern is more irregular. The vortices in zero applied field form an irregular stripe pattern following the irregular magnetic pattern, as shown in Fig. 3a. The location and the polarity of the underlying magnetic domains have been checked by local tunnelling spectroscopy performed at different applied fields of both polarities.

Now we turn our attention to the nucleation of the superconductivity close to *T*_c_ in zero applied external field. The order parameter should nucleate first at the location with minimum local stray field. However, if the magnetic domain size is too small, the order parameter cannot follow the rapid variation of the local field. In this case one expects overlapping of the superconducting nuclei, which produces a practically uniform superconducting state. We observe different behaviour for samples A and B. In sample A, with stripe width *w*≈200 nm, we detect a uniform nucleation of superconductivity when *T* is lowered below *T*_c_ (as shown in Supplementary Fig. 4). In sample B, with larger magnetic domains of *w*≈300 nm, the order parameter distribution is strongly modulated by the presence of the inhomogeneous field (Fig. 3 and Supplementary Fig. 5). Figure 3b shows a rescanned ZBC image in a smaller area acquired at *T*=2.0 K on sample B showing spontaneous vortices. The dashed square, 300 nm × 300 nm, represents the field of view used to directly visualize the nucleation of superconductivity. The ZBC map at *T*=5.5 K (Fig. 3c) shows the same conductance value everywhere, with local tunnelling spectra corresponding to the normal state. By decreasing *T* to 5.2 K, the ZBC map (Fig. 3d) shows areas of lower conductance localized along the white dashed line in Fig. 3b. This area corresponds to a region above the domain wall in the ferromagnet. The lateral size of the superconducting nucleus is ~120 nm. The value of the ZBC above the domain wall is only 15% lower than the conductance above the domains, due to *T* being very close to *T*_c_. The spatial evolution of the tunnelling spectra shows that the most superconducting region (lower ZBC) extends for 50 nm, with the ZBC gradually increasing on each side for about 35 nm. As *T* is further decreased to *T*=5.0 K (Fig. 3e) and *T*=4.88 K (Fig. 3f), the superconducting state becomes more robust, characterized by tunnelling spectra with a lower conductance at *E*_F_, and therefore, by more depletion of states at *E*_F_. A sequence of tunnelling spectra recorded along the dashed line in Fig. 3d appears in Fig. 3g.

## Discussion

To explain the different behaviour of the nucleation of superconductivity in the two samples we need to estimate the characteristic length scale of nucleation of the superconductivity. The criterion for domain wall superconductivity is *w*>2*ξ*(*T*_c_). The coherence length can be estimated from the upper critical field measured on a reference Pb film fabricated without ferromagnet. From the measured value of *H*_c2_(1.5 K)≈1,100 Oe we obtain *ξ*(*T*=1.5 K)≈54 nm, which yields *ξ*_0_=48 nm. The value of 2*ξ*(*T*=1.5ϰ) is consistent with the average diameter of vortices in our images^23^. Furthermore, this value of coherence length, very different from the bulk value of 87 nm^17^, is in agreement with values of 40÷50 nm reported for ultrathin Pb films^24^. From 
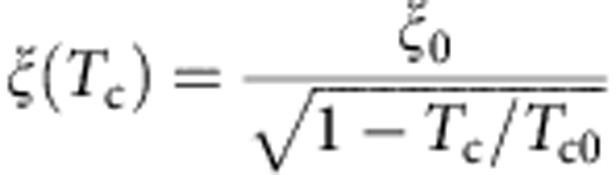
, where *ξ*_0_=48 nm is the zero temperature coherence length, *T*_c0_ is the critical temperature of the reference Pb film without ferromagnet and *T*_c_ is the superconducting temperature of the F/S bilayer, we obtain for sample A *ξ*(6 K)≈140 nm and for sample B *ξ*(5.5 K)≈110 nm. Therefore, *w*>2*ξ*(*T*_c_) in the case of sample *B* and *w*<2*ξ*(*T*_c_) in the case of sample *A*, which confirms that the two samples are in different regimes. Sample A is in the regime of overlapping superconducting nuclei above different domains; whereas in sample B the superconducting wave function is localized above regions with minimum stray field.

Finally, we turn our attention to the tunnelling spectra acquired at the domain wall at 5.2 K (Fig. 3c) and Supplementary Fig. 5(e)). The tunnelling spectra in the superconducting regions are much more smeared than the tunnelling spectra in the Pb reference film (deposited on an Al_2_O_3_ substrate; Supplementary Fig. 2). The increased broadening cannot be accounted for even considering the difference in *T*_c_ values. Lead is a strong-coupled superconductor. Therefore, the density of states should be obtained directly from the Eliashberg’s mean-field formalism, which accounts for recombination processes and electron–phonon scattering. However, a simpler approach was proposed by Dynes *et al.*^25^ where 
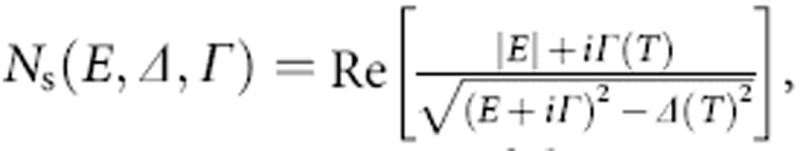
 with *N*_s_(*E*, *Δ, Γ*) being the density of states of the superconductor, Δ is the superconducting gap and *Γ* is the quasiparticle lifetime that takes into account all sources of scattering, including the orbital depairing produced by the stray field^26^. The experimental *dI*/*dV* spectra were fitted with the equation:


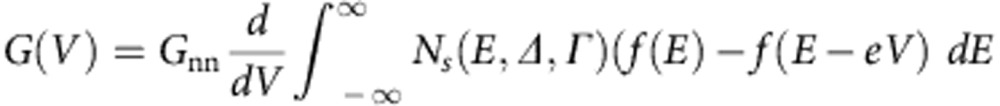


where, *f*(*E*) is the Fermi–Dirac distribution function, and *G*_nn_ is the conductance of the tunnel junction for high bias (normal state). This approach is widely used, since the values of *Δ* and *Γ* are in excellent agreement with theoretical predictions based on the Eliashberg formalism. In the case of Pb, Dynes’ density of states does not reproduce the experimental tunnelling conductance spectra acquired at low temperatures, due to the gap anisotropy^27^. Nevertheless, at higher temperatures, because of the thermal broadening, the anisotropy is usually smeared, and this approach provides reasonable fittings of the experimental data. For the spectra acquired above the domain wall region on sample B, at *T*=5.2 K (Fig. 3c and Supplementary Fig. 5(e)), the fitting yields values of *Δ*=0.7 meV and *Γ*=0.7 meV. For *T* in the range 5.2–5.35 K, the spectra do not show the presence of coherence peaks, but still present a dip around *E*_F_. These spectra cannot be fitted accurately with the above model; the fit yields a very high value of *Γ*, compared with the fits on the Pb reference film at the same reduced temperature. In the regime of domain wall superconductivity, the superconductivity is filamentary and the sample consists of superconductor-normal interfaces. Cooper pairs are squeezed into a very small volume, thus the quantum confinement in the transverse dimension becomes important and strongly modifies the wavefunctions. Thermal and quantum fluctuations^28^ will have a dominant role, and, therefore, suppress the superconductivity due to phase slips^29^. The lateral confinement also promotes an increase in the Coulomb repulsion. A description of the spatial evolution of tunnelling spectra in mesoscopic proximity systems in the presence of a non-homogeneous magnetic field is beyond any existing theory. New theoretical work would also have to take into account nonlocal effects in the normal regions due to electronic interference^30^. These results provide direct evidence of the possibility of order parameter manipulation in S/F hybrid systems and should stimulate theoretical work to take into account nonlocal effects in mesoscopic proximity systems.

## Methods

### Sample preparation

The experiments were conducted in a UHV low temperature STM combined with sample preparation for *in situ* fabrication of thin films (Unisoku USM-1300 and RHK electronics). The F/S heterostructures were fabricated in two different systems. The (Co-Pd) multilayers were deposited on Si substrates by direct current sputtering in the presence of an applied magnetic field in a dedicated system. The presence of the in-plane field during deposition favours a stripe-like magnetic domain pattern for the films as grown. A seed layer of 4 nm of Pd was used. The thickness of Co and Pd were fixed at 2 nm for a total of 200 repeats for sample A, and for a total of 50 repeats for sample B. This deposition is followed by a deposition of a 10-nm Al_2_O_3_ layer without exposure to air. The Al_2_O_3_ films were grown by radio frequency sputtering from an Al target at a rate of 0.2 nm s^−1^ in a partial pressure of oxygen. The gas ratios were 30 sccm Ar/10 sccm O_2_ (3:1). The hybrid F/S system is completed with the deposition of a 30 nm Pb thin film deposited in the UHV deposition chamber linked to the STM chamber. It is quite important that the Pb surface be kept in UHV to avoid contamination. The deposition of Pb was via e-beam evaporation at low temperature (120 K) followed by a room temperature annealing. This procedure enabled us to obtain flat films suitable for STM studies. Reference samples of Pb films of the same thickness were fabricated on an Al_2_O_3_ substrate and characterized for comparison.

### STM spectroscopy measurements

Tunnelling spectroscopy was performed using a standard lock-in technique with an alternating current modulation of 0.2 mV at 373 Hz. The conductance maps were acquired while scanning the tip over the sample surface at high voltage (20 mV), acquiring the lock-in signal at the Fermi energy at each location. Topography was always acquired simultaneously to assure the location where the spectroscopic information was recorded. All differential conductance spectra *dI*/*dV* were taken with the same tunnelling parameters with the junction stabilized at *V*=−10 mV, *I*=100 pA.

### Magnetic force microscopy

Magnetic force microscopy imaging was performed at room temperature using silicon cantilevers coated with a layer of Co–Cr alloy (MESP MFM probes by Bruker Corporation, Santa Barbara). These probes have a spring constant of 1–5 N m^−1^, a resonant frequency of 60–90 kHz, and a coercive field of nearly 400 Oe. Images were taken using a Bruker Multimode 8 atomic force microscope in the lift-mode regime at a lift height of 30 nm. Magnetic force microscopy in lift-mode proceeds typically in two steps. At first the topography of the sample is determined (one trace–retrace line), during which the cantilever tip is held at a short distance from the sample with the tip having constant frequency and drive amplitude, and keeping the amplitude of the cantilever oscillations the same by adjusting the tip distance from the sample surface. These measurements yield the topography line that includes any tilt and misalignment of the sample with respect to the horizontal plane. The measurement of the magnetic forces proceeds next as a second trace–retrace line along the same path of the first line. The cantilever completes the same path but retracted from the surface by a constant vertical offset distance (typically between 20–100 nm, and in our case 30 nm) with constant drive frequency and drive amplitude and without any feedback present. Since the atomic forces are short range and the magnetic interactions are long range, the second line scan provides information dominated by the magnetic tip–sample interaction. During the second line scan the phase of the cantilever oscillation is recorded. The contrast in this phase image is proportional to the change in the resonance frequency of the cantilever due to attractive or repulsive magnetic forces between the tip and the sample. Thus the phase image contrast is proportional to the changes in the local magnetization on the sample surface.

## Author contributions

M.I. and G.K. proposed the research project, M.I., J. F. and S.A.M., carried out the STM measurements and analysis of the STM data. V.N., S.A.M. and J.P. fabricated and characterized magnetic materials, G.K., S.T.C. and S.A.M. performed MFM measurements, S.A.M. fabricated the superconducting films. M.I., G.K., V.N. and S.D.B. wrote the manuscript. All authors discussed the results and contributed to the manuscript.

## Additional information

**How to cite this article:** Iavarone, M. *et al.* Visualizing domain wall and reverse domain superconductivity. *Nat. Commun.* 5:4766 doi: 10.1038/ncomms5766 (2014).

## Supplementary Material

Supplementary Information Supplementary Figures 1-5, Supplementary Notes 1-2 and Supplementary Reference
