# Supplementary material for: Visualizing domain wall and reverse domain superconductivity
Source: Nat Commun. 2014 Aug 28;5:4766. doi: 10.1038/ncomms5766 (PMC4354251; doi:10.1038/ncomms5766)
Supplement: Supplementary Information — Supplementary Figures 1-5, Supplementary Notes 1-2 and Supplementary Reference [file ncomms5766-s1.pdf]

Supplementary Information

Supplementary Figures

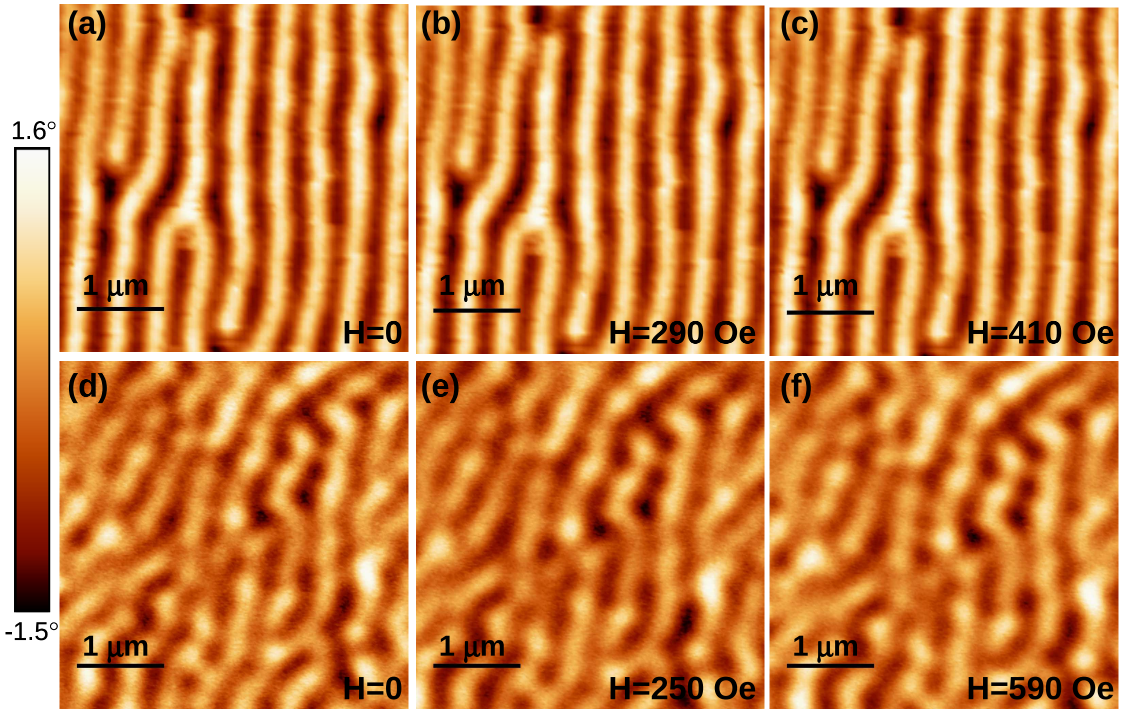

Supplementary Figure 1 | Magnetic domains: Room temperature 4 x 4 μm^2 MFM phase images, acquired in lift mode, of the (a), (b), (c) 200 bilayers of Co(2nm)/Pd(2nm) (Sample A) and (d), (e) , (f) 50 bilayers of Co(2 nm)/Pd(2nm) (Sample B) in external magnetic fields applied perpendicular to the surface. (a) *H*=0 Oe, (b) *H*=290 Oe, (c) *H*=410 Oe, (d) *H*=0 Oe, (e) *H*=250 Oe, (f) *H*=590 Oe. Scale bars are 1 µm. MFM images have been acquired after deposition of the Al_2O_3 layer.

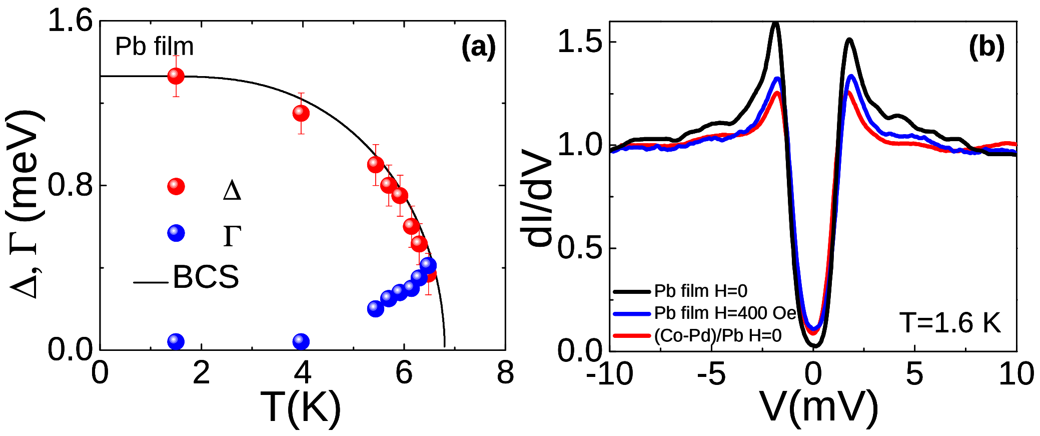

Supplementary Figure 2 | Comparison between reference Pb film and (Co-Pd)/Pb systems: (a) Temperature dependence of the gap (red dots) and of the quasiparticle lifetime Γ (blue dots) for the reference 30 nm Pb film, derived by the BCS DOS fitting with the Dynes formula, as described in the main text. The error bars represent the standard deviation obtained from the fit. The continuous black line is the BCS gap equation corresponding to T_c=6.8 K. (b) Comparison between the tunnelling dI/dV spectrum on the Pb reference film at T=1.6 K in zero applied magnetic field, a tunnelling dI/dV spectrum acquired on the same film at the same temperature in a magnetic field H=400 Oe applied perpendicular to the film surface and a tunnelling dI/dV spectrum acquired on a (Co-Pd)/Pb (Sample B) system in a zero applied external field. In all cases spectra have been acquired in locations far from vortices and with the same tunnelling conditions: V=-10 mV, I=100 pA. The dI/dV curves have been normalized to the value of conductance at V=-10 mV.

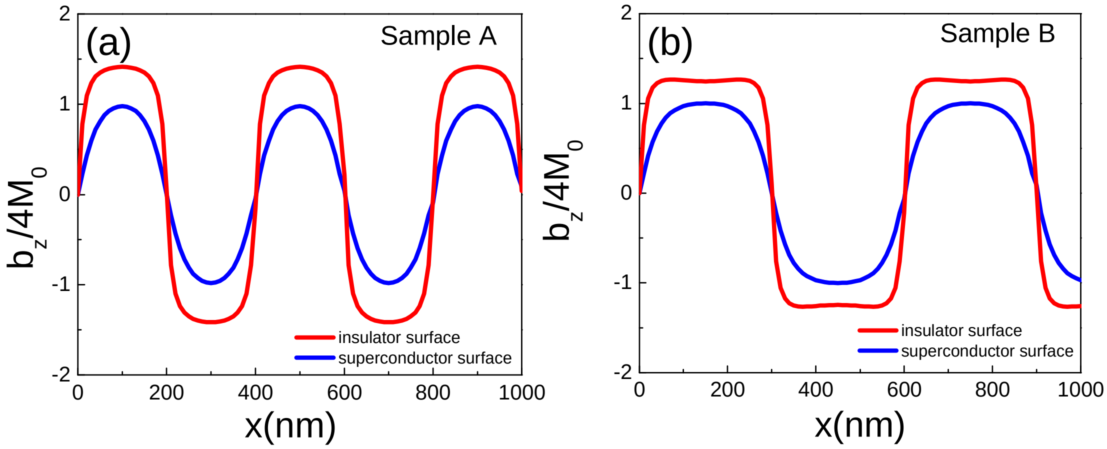

Supplementary Figure 3 | Stray Field Profiles in (Co-Pd)/Pb systems: Calculated magnetic field distribution for the Ferromagnet-Insulator-Superconductor systems for (a) Sample A (ferromagnet thickness D=800 nm, magnetic domain width w=200 nm) and for (b) Sample B (ferromagnet thickness D=200 nm, magnetic domain width w=300 nm). In both graphs the red line represents the stray field at the top of the insulator's surface (at a distance z=10 nm from the ferromagnet's surface) and the blue line is the magnetic profile at the surface of the superconductor (at a distance z=40 nm from the surface of the ferromagnet). This calculation is based on the formula given in Supplementary note 1.

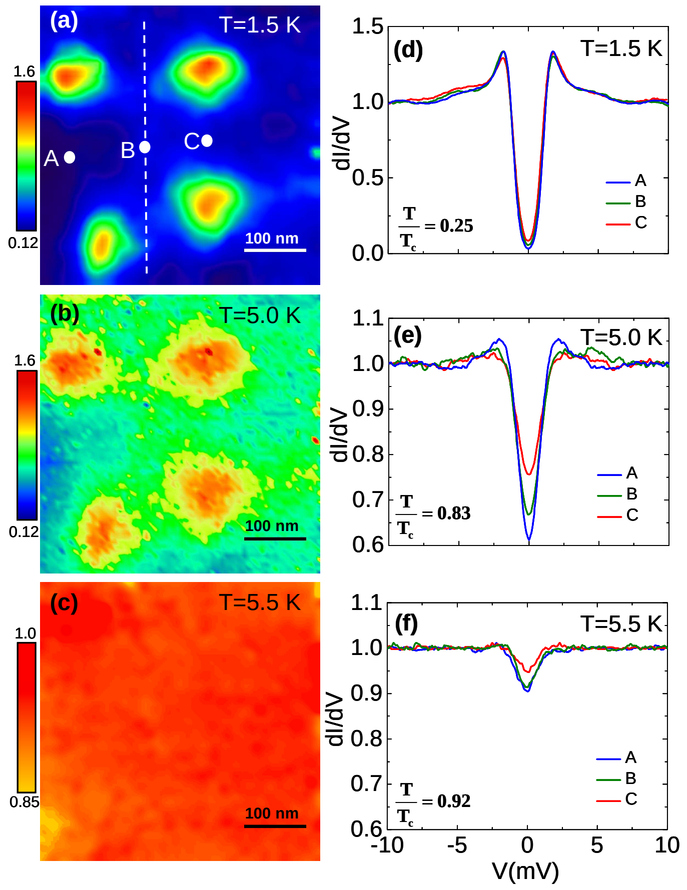

Supplementary Figure 4 | Emergence of superconductivity in the (Co-Pd)/Pb Sample A: (a), (b), (c) LDOS maps at the Fermi energy acquired at H=0 Oe and at (a) T=1.5 K, (b) T=5.0 K, (c) T=5.5 K. All maps have been normalized to the maps acquired simultaneously at V=-10 mV. The scan areas in (a), (b) and (c) are 450 x 450 nm^2 and the scale bars are 100 nm. The white dashed line in (a) indicates the approximate positions of the domain walls as inferred from the vortex configurations at different fields (Fig. 2 in the main text).The graphs in (d), (e) and (f) show the tunnelling spectra acquired at different location across the domain wall (Positions A, B, C) of the corresponding LDOS map taken at the same temperature. All spectra have been acquired with the same tunnelling conditions V=-10 mV and I=100 pA and normalized at V=-10 mV.

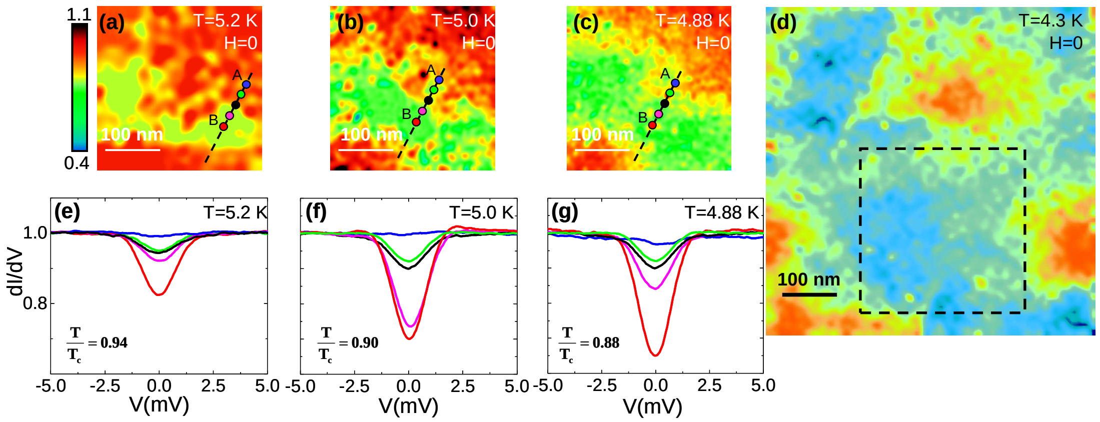

Supplementary Figure 5 | Emergence of superconductivity in the (Co-Pd)/Pb Sample B: (a), (b), (c), (d) LDOS maps at the Fermi energy acquired at *H*=0 Oe and at (a) *T*=5.2 K, (b) *T*=5.0 K, (c) *T*=4.88 K and (d) *T*=4.3 K. All maps have been normalized to the maps acquired simultaneously at *V*=-10 mV. The colour scale (representing the normalized zero bias conductance) is the same for all images. The scan areas are 300 x 300 nm^2 for (a), (b), (c) and 600 x 600 nm^2 for (d). The maps in (a), (b) and (c) have been acquired at the location indicated with a dashed square in (d). The graphs in (e), (f) and (g) show the tunnelling spectra acquired at different location across the domain wall (along the dashed line from A to B every 15 nm) of the corresponding LDOS map at the same temperature. The colours used for the tunnelling spectra are the same used to indicate the locations where the spectra were acquired (coloured dots on the corresponding LDOS map). All spectra have been acquired with the same tunnelling conditions *V*=-10 mV and *I*=100 pA and they have been normalized to the value of conductance at *V*=-5 mV.

Supplementary Note 1

Magnetic Field Profile in Ferromagnet-Insulator-Superconductor Systems

The distribution of stray fields for a stripe domain structure in a ferromagnetic film can be calculated analytically. If the film is parallel to the *xy* plane the *z* component of the stray field in the direction perpendicular to the film surface is[1]:

$$b_{\mathbf{z}}(x,z) = \mathrm{Im}\left[4M_{\mathbf{0}}\left[\ln\tan\frac{\pi t}{2w} - \ln\tan\frac{\pi(t-iD)}{2w}\right]\right] \quad (1)$$

where $t = x + iz$, D is the ferromagnet thickness and w is the stripe domain width. In a Ferromagnet-Insulator-Superconductor system if the London penetration depth is larger than the superconductor thickness the stray field is almost not affected by the presence of the superconductor and Supplementary Equation (1) can be used to calculate the stray field at the top of the superconductor's surface. In Supplementary Fig. 3 the stray field profiles for Sample A and Sample B are reported at different distances *z* from the ferromagnet's surface.

Supplementary Note 2

Spatial variation of the local density of states in (Co-Pd)/Pb systems

In Supplementary Fig. 4 and Supplementary Fig. 5 we describe the local variations of tunnelling spectra in the (Co-Pd)/Pb Sample A and B respectively. In Supplementary Fig. 4(a), (b), and (c) we report the LDOS at the Fermi energy acquired at different temperatures across the superconducting temperature T_c. In Supplementary Fig. 4(d), (e) and (f) we report typical conductance spectra acquired across the domain wall. In Sample A the spectra across the domain wall are very similar with very small changes of the ZBC values. In particular very close to the critical temperature at T=5.5 K (i.e. T/T_c=0.92) the

sample is already in the superconducting state with tunnelling spectra that show coherence peaks and a value of ZBC very consistent in the field of view ranging from 0.9 to 0.94 (Supplementary Fig. 4(c) and Supplementary Fig. 4(f)). In Supplementary Fig. 4(c) the LDOS map at the Fermi energy shows a very uniform state. We do not observe on this sample the variation in ZBC that are observed instead in Sample B at similar values of reduced temperature. In this case, indeed, as explained in the main text the narrow dimension of the magnetic domains favours the overlap of the superconducting nuclei across the magnetic domains and a more homogeneous superconducting state emerges.

In Supplementary Fig. 5 we report the LDOS maps at the Fermi energy (Supplementary Fig. 5 (a), (b), (c) and (d)) and local tunnelling spectra dI/dV acquired at different temperatures on Sample B (Supplementary Fig. 5(e), (f) and (g)). The local spectra acquired across regions above two stripes of opposite polarities show that at T=5.2K (*i.e.* T/T_c=0.94), there is a strong variation of local spectra with ZBC values ranging from 0.82 to 1 (Supplementary Fig. 5(a) and (e)) as opposed to Sample A. By reducing further the temperature at T=5.0 K (*i.e.* T/T_c=0.90), the ZBC values are still inhomogeneous with values ranging from 0.7 to 1 (Supplementary Fig. 5(b) and (f)). At T=4.88 K (*i.e.* T/T_c=0.88), the ZBC values range from 0.65 to 1 (Supplementary Fig. 5(c) and (g)). Finally at T=4.3 K, we can clearly distinguish single vortices along each magnetic domain. Therefore, in Sample B while decreasing the temperature below T_c the superconducting order parameter nucleates first at the regions above the domain wall.

Supplementary References

1. Sonin, E. B. Comment on Ferromagnetic film on a superconducting substrate. *Phys. Rev.B* **66**, 136501 (2002).
